# The added value of establishing a lexicon to help inform, compare, and better understand the implementation of policy, systems, and environmental change strategies in Supplemental Nutrition Assistance Program Education

**DOI:** 10.1016/j.pmedr.2019.100873

**Published:** 2019-04-25

**Authors:** Jack Thompson, Katherine Sutton, Tony Kuo

**Affiliations:** a Division of Chronic Disease and Injury Prevention, Los Angeles County Department of Public Health, Los Angeles, CA 90010, USA; b Department of Epidemiology, UCLA Fielding School of Public Health, Los Angeles, CA 90095, USA; c David Geffen School of Medicine at UCLA, Los Angeles, CA 90024, USA; d Population Health Program, UCLA Clinical and Translational Science Institute, Los Angeles, CA 90095, USA

**Keywords:** SNAP-Ed, Supplemental Nutrition Assistance Program Education, LAC, Los Angeles County, PSEs, Policy, systems, and environmental change strategies, USDA, United States Department of Agriculture, LHDs, Local Health Departments, FPL, Federal Poverty Level, CHIS, California Health Interview Survey, Obesity prevention, Public health, Policy, systems and environmental change, Lexicon, Program implementation, Program evaluation

## Abstract

Categorization of terms/concepts/constructs that allows for better understanding and comparison of public health interventions is often lacking in program implementation and evaluation. A classification system such as a lexicon, when used appropriately, can help address this need. The present narrative describes a lexicon of policy, systems, and environmental change strategies (PSEs) that was developed and prototyped to aid local implementation of Supplemental Nutrition Assistance Program Education (SNAP-Ed) interventions in obesity prevention. The lexicon was reviewed and refined by a panel of experts who provided iterative feedback on the system's scope and utility. To develop the lexicon, a team from the local health department: (i) conducted an inventory (community context scan) of SNAP-Ed PSEs implemented in Los Angeles County during 2010–2015; (ii) assessed commonalities among PSEs that were translated into “index factors” to contextualize terms/concepts/constructs relevant to SNAP-Ed services planning; and (iii) convened a panel of experts to review and test the classification system for quality and usability. In the latter activity, the panel reviewed the terms/concepts/constructs within the context of two geographical areas and by the selected PSEs. The final version of the lexicon organized the terms/concepts/constructs of the local SNAP-Ed PSEs into overarching categories, so they can be compared/assessed by type, content, and/or impact. The goal of the project was to create a classification system that can help facilitate meaningful communications among program implementers, evaluators, and community stakeholders. The lexicon has practical implications and potential applications for other jurisdictions interested in reducing obesity rates through SNAP-Ed PSEs.

## Highlights

• Policy, systems, and environmental changes (PSEs) in prevention can vary widely.
• Comparison of PSE strategies is limited by a lack of standard categorization.
• A lexicon can improve communications among PSE implementers and evaluators.
• A lexicon can enhance data comparability and help shape PSE designs/evaluations.

## 1 Introduction

Public health practice often requires a systematic process to assure quality data collection, interpretation, and dissemination of results (Hall et al., 2012). Standardized terminology leading to a clearer understanding of application nuances for public health interventions is essential for successful implementation of these strategies in diverse settings (Kindig, 2007). This classification approach is basic to human nature and serves as a foundation for defining and communicating terms, concepts, and/or constructs (e.g., reasoning, language, statistics) in science (Bailey, 1994). A “lexicon” is a particular categorization or information management technique that is used to create standardized vocabulary for a particular subject matter or topic area (Merriam-Webster Dictionary, 2017). Its intended result is an index of terms with common definitions or constructs (“index factors”) that are universally comparable and can be communicated among program implementers, evaluators, and community stakeholders alike. Seamless communication of terms/concepts/constructs among key actors of successful program or policy implementation is an operational gap that is often overlooked in health and public health practice.

The lexicon developed and presented in this narrative is specific to public health interventions focused on obesity prevention, a priority area that is well-characterized in the literature (Bunnell et al., 2012; Robles et al., 2014; Wang and Beydoun, 2007; Wu et al., 2017). The narrative describes the process of creating a classification system that is strengthened by iterative refinements – i.e., pilot testing of terms/concepts/constructs using a modified Delphi method. The narrative describes the development and utilization of a lexicon, tailored to classifying local strategy interventions that were featured in the Supplemental Nutrition Assistance Program Education (SNAP-Ed) projects in Los Angeles County (LAC) during 2010–2015. The Los Angeles County Department of Public Health (DPH) utilized this lexicon to systematically categorize policy, systems, and environmental change strategies (PSEs) that were implemented in the field during the sampled time period. PSEs that were examined included a broad range of interventions, ranging from healthy food and beverage standards to establishment of community gardens to corner-store makeovers to adoption of school wellness policies. The overall goal of the project was to create a classification system that can help facilitate meaningful communications among program implementers, evaluators, and community stakeholders under varying local conditions.

## 2 Methods

### 2.1 Context

With passage of the *Healthy, Hunger-Free Kids Act*, the federal United States Department of Agriculture (USDA) SNAP-Ed program adopted a socioecological framework to guide service delivery of nutrition education and related health promotion resources (McLeroy et al., 1988). A benefit of this structure is that it gave local programs “greater flexibility to include environmental interventions and policy level work that complemented nutrition education and health promotion” (Funding for SNAP-Ed, 2017). Under this law, local health departments (LHDs) in California had increased autonomy to plan and tailor interventions to the needs of local communities. This resulted in the implementation of varying combinations of PSEs that addressed social and demographic conditions known to affect obesity risk and food security in the region (Kuo et al., 2016).

During the latest SNAP-Ed funding cycle (2013–2016), planners of the Los Angeles County effort recognized, early on, that a common language describing the SNAP-Ed interventions may be necessary to facilitate meaningful communications and coordinated actions among implementers, evaluators, and community stakeholders. They understood that such a classification system (i.e., a lexicon) should be constructed based on data and information from a landscape analysis of regional policies and programming before and after the start of the latest cycle of SNAP-Ed funding, an accurate assessment of population health burden, and qualitative input from experts who have extensive knowledge and experience in implementing PSEs.

### 2.2 Lexicon development

To develop the lexicon, the project team utilized data derived from several program sources, including: (1) a community context scan, (2) a health indicators project that synthesized health data by geographical region, and (3) a convening of an expert panel, using the modified Delphi method to pilot test and refine the classification system. The Delphi method is an iterative process framework based on multiple rounds of queries, typically asking experts to reach consensus or to explore areas of future thinking that goes beyond what is currently known (RAND Corporation, 2019). The iterative process can be adapted (‘modified’) for use in a wide range of environments. This adapted approach is often called a “modified Delphi.”

The lexicon groupings were referred to as “index factors” in this project, representing observed commonalities or identified patterns of PSEs that are organized or contextualized in a meaningful, consistent way. To group and assign terms/concepts/constructs to these index factors, data were obtained and synthesized for four key SNAP-Ed PSE strategy interventions implemented in two of the eight Service Planning Areas (SPAs) in LAC –Metropolitan (SPA 4) and South Los Angeles (SPA 6). Both SPAs covered geographic areas where there were high densities of low-income, SNAP-Ed eligible households. SNAP-Ed eligibility is determined primarily based on census tracts where at least 50% of the population have income at or below 185% of the Federal Poverty Level (FPL). The four selected interventions (focus areas) included: *healthy food and beverage standards*, *community gardens*, *healthy retail makeover*, and *farmers markets*.

(i) *Community context scan*

The community context scan, developed in partnership with Ad Lucem Consulting, included an initial review of relevant obesity prevention efforts in Los Angeles County during 2010–2015. The resulting database cataloged obesity prevention activities in the region for the past 5 years. It included information derived from a series of 51 key informant interviews with leaders of various sectors such as academia, non-profit, government, community clinics, and the private sector (e.g., insurance providers). Special focus was placed on capturing a snapshot of target areas of SPAs 4 and 6 where many residents were poor and were eligible for SNAP-Ed services. The context scan provided qualitative and quantitative data that aided the development of the lexicon, including information on type, setting, reach, factors associated with program implementation, and sustainability of key PSE strategy interventions. The scan was not an exhaustive review of all PSEs in LAC during 2010–2015.

(ii) *Health indicators project*

Population-level health indicators for SPAs 4 and 6 were assessed in parallel to the community context scan during the 2010–2015 time period using the California Health Interview Survey (CHIS). CHIS data were broken down by SPA designations; for comparison, data for SPA designations other than 4 and 6 were also included. To explore differences in the health indicator by selected geography and demographics, several statistical analyses were conducted to describe the associations between the indicators and known risks of obesity and nutrition-related diseases (e.g., poor diet, physical inactivity). When feasible, these indicators and other existing sources of data were used to inform and select key terms/concepts/constructs for inclusion in the lexicon. For example, data sources such as the *Communities of Excellence in Nutrition, Physical Activity, and Obesity Prevention* (CX3) project contained pertinent neighborhood-level data that were acquired using the recommended community assessment tool developed by the California Department of Public Health. In combination, this information provided context and contributed to the methodology that was used to categorize and operationalize the health elements (terms and constructs) of SNAP-Ed interventions and services implemented in LAC.

(iii) *Panel of experts*

Using a modified Delphi method, the lexicon categories that were derived from the community context scan and the health indicators project were further contextualized with input from a panel of experts convened by DPH. The panel of experts (there were four of them) comprised members with expertise in PSE implementation and community design. As a condition of participation and to preserve confidentiality, each member's organizational affiliation(s) were not disclosed. These experts' input allowed for selective testing/prototyping of terms/concepts/constructs identified in the first two phases of the lexicon development process. The overall Delphi process took approximately 6 months to complete – two rounds of surveys, one group discussion, and the synthesis of data gathered.

## 3 Results

Using the community context scan results (Table 1, Table 2), and the corresponding health indicators project data (Table 3), the project team generated eight index factors that make up the lexicon classification system (Table 4). These eight factors (or domains) included: general thematic area (nutrition versus physical activity), specific strategy intervention, setting, type of implementing organization, reach (of target group), level of PSE access, outcome(s) desired by program participant, and opportunities for PSEs to encourage change.

**Table 1:** Policy, systems, and environmental change strategies (PSEs) implemented in Los Angeles County during 2010–2015.

Characteristics of PSEs (including type) assessed in the Community Context Scan, 2010–2015
• Type of organization • Specific PSE strategy • Target geography • Population • Ethnicity of target/Priority populations • Socioeconomic status • Primary language spoken • Program/Initiative goals and objectives • Outcomes achieved

**Table 2:** Number and type of implemented PSEs in Los Angeles County, as captured by the Community Context Scan, 2010–2015.

Number and type of SNAP-Ed PSE strategy interventions implemented in Los Angeles County^a^	
SNAP-Ed strategy interventions	Number implemented^b^
1. Efforts in Child Care Centers	14
2. Wellness policies	9
3. Farm to school/fork	4
4. Joint/Shared use agreements	7
5. Healthy retail	24
6. Restaurants/Mobile vending	14
7. Physical Activity programs	24
8. Gardens	15
9. Worksite program	4
10. Active transport	23
11. Farmers markets	20
12. Healthy food and Beverage standards	27
13. Healthy food and Beverage availability^c^	56
*Other common strategy area: breastfeeding*	6
*Other common strategy area: parks*	26

a Estimated at the time of the Context Scan data collection during 2014–2015.

b Partner organizations may have addressed or implemented more than one PSEs. Thus, some of these strategy interventions may have been double counted in the table.

c Ad Lucem Consulting interpreted the SNAP-Ed interventions more broadly than currently defined (i.e., to include the availability of healthy foods and beverages at venues beyond simply community events): “Collaborate with local youth-serving organizations working with low-income populations (such as parks and recreation, sports leagues, booster clubs, etc.) to ensure that healthy foods and beverages are available at community events for purchase. Encourage organizations to seek healthy beverage sponsorships.”

**Table 3:** Health indicators by Service Planning Area (SPA) in Los Angeles County.

Health indicators	Service Planning Area (SPA)								
	Los Angeles County	SPA 1	SPA 2	SPA 3	SPA 4	SPA 5	SPA 6	SPA 7	SPA 8
Diabetes (%)	10.4	7.9	8.1	12.1	9.7	7.2	12.3	11.2	12.4
Hypertension (%)	27.4	27.8	22.9	25.9	31.3	25.3	30.3	25.5	32.9
Overweight (%)	35.7	33.7	36.7	37.3	37.7	29.5	38.5	33.8	34.0
Obese (%)	26.3	27.1	23.6	22.4	22.5	17.7	40.2	32.7	27.7
Fast food 2+ times per week (%)	43.6	48.9	42.1	42.5	34.8	29.0	43.9	51.2	46.0
One or more sugar-sweetened beverages per day (%)	15.2	21.3	13.2	13.5	12.6	8.7	23.9	18.4	15.6
Walked for transport (%)	55.7	41.4	52.8	48.6	71.5	60.3	62.2	54.8	54.3
Walked for leisure (%)	64.9	67.6	65.8	64.7	62.9	71.4	64.3	63.6	63.1
SNAP-Ed eligible	43.2	41.0	39.5	40.7	56.8	16.2	61.8	48.6	37.9
SNAP recipient	12.5	32.6	9.4	8.7	16.1	4.8	15.4	15.1	9.5

*SNAP-Ed eligible* is defined as being low income (below 185% of the Federal Poverty Level) or enrolled in Medi-Cal.

**Table 4:** Groupings (index factors) of the lexicon: Categorization of policy, system, and environmental change strategies (PSEs) in Supplemental Nutrition Assistance Program Education (SNAP-Ed), Los Angeles County, 2010–2015.^a^

Groupings (index factors)	Thematic area	Specific PSE strategy area	Setting	Type of intervention	Reach	Access	Use/Utility	Opportunities to inform/encourage change
Description(s)	*Nutrition* versus *physical activity*	• *Healthy retail* • *School wellness policy* • *Restaurants* • *Mobile vending* • *Institutional food* • *Farmers markets* • *Gardens* • *Farm to school/fork* • *Food/Beverage policy* • *Joint/Shared use* • *Active transport* • *Worksite*	*Community; School; Child or Adult Care; Health Care; Worksite; Faith-Based*	*Policy (organizational); Policy (regulatory); Policy (school); Policy (internal government agency); Systems (practices); Environment (physical changes)*	*Small organization (<20 employees); Large organization (>20 employees); Neighborhood; City; County; Regional (*e.g.*, Southern California); State; Federal*	*Facilitate access to (new) environment/infrastructure; Establish new environment/infrastructure; Add amenities or improve environment/infrastructure*	*Create programs to attract use; Market and advertise; Promote social cohesion*	*Signal innovation; Codify a concept; Stimulate change/implementation*

a While the intent of the lexicon is to inform SNAP-Ed program implementation and evaluation, not all strategies were included in the lexicon development process. The final version of the lexicon allowed for iterations and tailoring of the categories (or additional categories) to program circumstances, depending on the particular strategy intervention being implemented.

In the convening of the panel of experts, the terms/concepts/constructs derived from these first two phases of lexicon development were pilot tested and further refined for quality and usability – i.e., how the terms/concepts/constructs should be interpreted and applied in real world situations; under which context should they be used to plan and/or inform action (Table 5, Table 6). As an example, during the modified Delphi surveys and discussion, the panel was asked to specifically describe and rate the level of difficulty in “establishing” and/or “sustaining” strategy interventions in the two selected SPAs (4 and 6) in LAC. Two issues that were raised during this query included: (a) time/duration (e.g., months or years required for an intervention to achieve health impact) and (b) intervention strength (e.g., rates of uptake, spread, organizational capacity, community readiness, availability of funding to support or to carry out the work in the surrounding communities, and how well each intervention is/was received by the intended audiences).

**Table 5:** Modified Delphi method results, used to inform further lexicon refinement.

Expert panel ratings^a^				
PSE estimates for target geographies	Community gardens	Healthy food/beverages standards	Healthy retail	Farmers markets
SPA 4 – Estimated level of difficulty (rating) for **establishing** the PSE?	5	5.5	6.5	7.25
SPA 4 – Estimated level of difficulty (rating) for **sustaining** the PSE?	7.25	5	8.25	7
SPA 6 – Estimated level of difficulty (rating) for **establishing** the PSE?	5.25	5	7.25	7.5
SPA 6 – Estimated level of difficulty (rating) for **sustaining** the PSE?	6.5	4.5	8	7.75

a Based on a scale of 1–10, with 1 being the least difficult and 10 being the most difficult. Index factors or concepts (terms, constructs) that were considered are further described in Table 6.

**Table 6:** Three examples of policy, systems, and environmental change (PSE) terms/concepts/constructs that were pilot tested or prototyped by a panel of experts, using the modified Delphi method.

Strength	To determine overall “strength” of an intervention, the expert panel was asked to estimate a rating for both **Establishing** and **Sustaining** the selected PSEs presented. Various factors were considered including “rate of uptake” and “spread” of the selected strategies in the two high needs SPAs (4 and 6). Understanding the various intervention components (i.e., readiness/start-up factors, scalability, funding availability, organizational readiness, sustainability) for the selected PSEs helped to contextualize the lexicon terminology and constructs. The ratings helped set realistic expectations about the timing, content, and implementation of the selected interventions.
Rate of uptake	This concept refers to an estimate, based on experience, of how quickly an intervention can be adopted and implemented for any given PSE effort. The rate can be recorded as an increment of time (e.g., took XX months, took XX years). Factors that were considered included level of difficulty in establishing and sustaining the intervention (e.g., organizational or community readiness, lack of or availability of funding support to carry out the work in the surrounding communities, how well an intervention is/was received by the intended audiences).
Spread	This concept refers to an estimate, based on experience, of the distribution or receptivity of PSE strategies or programming in the field or in a given community/region. Estimate or documentation of spread often correlated with the impact or success of a program or PSE effort in a community based on how widely the intervention was adopted.

The results of the modified Delphi allowed the project team (representing the SNAP-Ed project in LAC) to fine-tune several of the terms/concepts/constructs in the lexicon. For example, “intervention spread” became more multi-dimensional with added complexity based on inputs from the panel. The term or concept was ultimately defined as the *distribution or receptivity of PSE strategies or programming in the field or in each community/region that may correlate with the reach and/or impact of a program or PSE activity in that community*.

Because the overarching categorization approach was not intended to be exhaustive (i.e., not all PSEs were inventoried and included in the context scan during 2010–2015), they should be interpreted within the context of continuous learning and application of best practices using data from the most available evidence base. The lexicon ultimately serves as a starting point for facilitating a more standardized dialogue among program implementers, evaluators, and community stakeholders; these groups are responsible for making the various SNAP-Ed interventions a success across the county. It has been estimated that during 2010–2015, >150 PSE strategy interventions were successfully implemented throughout LAC (Table 2).

## 4 Discussion

Despite consensus on how to improve nutrition and related health behaviors, little is known about which mix of strategies is optimal for preventing these obesity risk factors in different settings (Bunnell et al., 2012; Gillespie et al., 2015). This lack of clarity in public health practice is in part due to the fragmented understanding of the various terms/concepts/constructs often used to describe PSEs and their complexities (McQueen, 2000). Compounded by ambiguous definitions or descriptors of PSEs in the current literature, a knowledge gap exists in program implementation and evaluation; this gap can lead to inconsistent interpretation of intervention effects and impede program progress. The present project attempted to establish and use a lexicon to aid SNAP-Ed PSE implementation, thereby reducing this gap in public health practice.

Systematic naming or classification of intervention parameters and related index factors can provide greater opportunities to standardize data collection and analysis. Over time, having commonalities in information management and synthesis terminology can lead to more reliable and accurate longitudinal tracking of progress in program implementation. This in turn can lead to better designs of program improvement efforts; not to mention greater confidence in the validity of the information being gathered, communicated, and used. More specific intervention descriptors and classification protocols can enhance surveillance and allow for greater ease in replicating interventions because of reduced guesswork in the definition and application of what is/are effective in a specific community. The capacity to generate and use this type of iterative process can, if done meaningfully, help lower the overall cost of intervention selection and implementation, and may help facilitate quicker diffusion of innovations (Rogers, 2003).

The lexicon is intended to address these and other issues about program implementation and evaluation. By categorizing a broad range of factors that could be considered within each PSE type, the system ultimately aims to help program implementers, evaluators, and community stakeholders communicate their vision and roadmap for program implementation, using a consistent vocabulary to describe, execute, and evaluate PSEs. The need for clear delineations within and between the selected strategy interventions was apparent during the modified Delphi discussion. The panel of experts was given an opportunity to pilot test or prototype (i.e., review for quality and explore the system's possible uses) several key terms/concepts/constructs within the context of implementation nuances for each of the selected strategies. The resulting inputs were invaluable for refining the lexicon – e.g., variations among communities around safety, gentrification, organizational capacities, political will, and density of eligible populations within eligible census tracts became added considerations in the construction of the system.

The present narrative describes the development and refinement of a lexicon that can be used to better understand PSEs in their “real world” context, especially as they relate to the growing obesity epidemic in LAC. The classification system (a tool) is proposed as an iterative approach for program planning and implementation, with built-in flexibility for ongoing adjustments and updates. By organizing PSEs into lexicon categories, the present project sought to establish a usable framework for improving local program communication and implementation of SNAP-Ed strategies. Future efforts should consider adapting and optimizing this lexicon for use by other LHDs that are interested in streamlining or improving SNAP-Ed delivery across the United States.

## 5 Conclusions

Public health practice is driven by an ongoing process of systematic surveillance, data collection, analysis, and interpretation of results. The value of these outputs depends on their comparability, application, and how they are communicated among program implementers, evaluators, and community stakeholders. Current PSE categories are generally too broad or amorphous to allow for meaningful, consistent comparisons or documentation of program progress/impact. A lexicon on intervention type, content, and impact, based on agreed upon terms/concepts/constructs, can help address this gap in public health practice. During a time of heightened scrutiny of public health programs, the need for meaningful categorization of program interventions is critical. In the case of SNAP-Ed in LAC, a lexicon of PSE strategy interventions appeared to confer added value for addressing this accountability process in social and health services delivery.

## Disclosure

The authors report no financial disclosures or conflicts of interest.
